# ESBR Nanocomposites Filled with Monodisperse Silica Modified with Si747: The Effects of Amount and pH on Performance

**DOI:** 10.3390/polym15040981

**Published:** 2023-02-16

**Authors:** Lijian Xia, Anmin Tao, Jinyun Cui, Abin Sun, Ze Kan, Shaofeng Liu

**Affiliations:** 1 Key Laboratory of Biobased Polymer Materials, Shandong Provincial Education Department, School of Polymer Science and Engineering, Qingdao University of Science and Technology, Qingdao 266042, China; 2 State Key Laboratory of Marine Coatings, Marine Chemical Research Institute Co., Ltd., Qingdao 266072, China

**Keywords:** monodisperse silica, latex compounding technique, Si747, ESBR/silica nanocomposites

## Abstract

To prepare silica/rubber composites for low roll resistance tires, a novel strategy was proposed in this study, in which autonomous monodisperse silica (AS) was prepared and modified using 3-mercaptopropyloxy-methoxyl-bis(nonane-pentaethoxy) siloxane (Si747), after which silica/emulsion styrene butadiene rubber (ESBR) master batches were produced using the latex compounding technique. Meanwhile, the commercial precipitated silica (PS) was introduced as a control. In this study, the effects of amount of Si747 and pH value on the properties of the silica/ESBR composites were systematically analyzed. Thermal gravimetric analysis (TGA) and Fourier transform infrared (FTIR) results indicated that Si747 reduced the silanol group by chemical grafting and physical shielding, and the optimum amounts of Si747 for AS and PS modification were confirmed to be 15% and 20%, respectively. Under a pH of 9, ESBR/modified AS (MAS) composites with 15% Si747 presented better silica dispersion and a weaker Payne effect, compared with ESBR/modified PS (MPS) composites with 20% Si747. Meanwhile, in terms of dynamic properties, the ESBR/MAS composites exhibited a better balance of lower rolling resistance and higher wet skid resistance than the ESBR/MPS composites.

## 1. Introduction

Silica is an extremely important reinforcing filler in the rubber industry [1,2,3]. Therein, silica/styrene butadiene rubber (SBR) composites are usually used for green tires because silica provides a much better combination of low rolling resistance and considerably high wet skid resistance than composites of carbon black fills [3,4,5]. However, silica features high polarity and strong hydrophilicity because numerous hydroxyl groups exist on the silica surface, resulting in serious aggregation and poor compatibility with non-polar rubber and poor dispersion in the rubber matrix [6,7,8]. In order to improve the dispersion of silica, one major method is to consume or shield the hydroxyl groups and form a “coupling bridge” between the silica and the rubber by introducing different kinds of silane coupling agent [9,10,11]. Among them, *bis*(3-triethoxy-silylpropyl) tetrasulfide (TESPT, Si69) is the most commonly used. Li used a “two-step method” to investigate the modification process and elaborated the modification mechanism in detail based on the traditional method of blending rubber, silica and Si69 to prepare compound [12].

The latex compounding technique is another common method developed for improving silica dispersion and lowering the processing temperature in traditional mechanical blending. Many attempts have been made to explore feasible and advantageous technologies using the latex compounding technique, including preparing in situ and then co-flocculating silica particles using the sol–gel method in rubber latex [13,14,15], or mechanical stirring of commercial silica slurry followed by blending with rubber latex [16].

In our previous report [17], we prepared autonomous monodisperse silica (AS) via the sol–gel method and precisely controlled the morphology and particle size, then prepared Natural Rubber (NR)/AS master batches via the latex compounding technique. Meanwhile, commercial precipitated silica (PS) was introduced as a control. NR/AS composites exhibited better silica dispersion and weaker filler–filler interactions compared to NR/PS composites. However, it should be noted that the process of modifying AS and PS with Si69 was inefficient and time-consuming, and Si69, due to its abundant polysulfide bonds, can more easily cause scorching of rubber during the compounding process along with poor storage safety. Moreover, the amounts of ethanol aggravated volatile organic compounds (VOC) emission. In summary, the research and development of silane coupling agents with low sulfur (*Bis*(triethoxysilylpropyl)disulphide, TESPD, Si75, Si266) [18,19] or shielding sulfur (Octanoyl thioester-protected mercaptosilane, NXT) [20] are of great interest. Both Si75 and NXT decrease reactivity and ensure process safety; NXT also reduces VOC production and improves the rolling resistance of compounds. Due to the strict requirements of EU labeling law for VOC and the higher price of NXT, a kind of silane coupling agent where the long-chain polyalkyl ether alcohols on the silane’s silicon atom replace the ethoxyl is used instead, resulting in greatly reduced VOC emission. In this case, long-chain polyalkyl ether alcohols can shield the reactive sulfhydryl group and delay the activation of the accelerant and sulfur. In addition, long-chain polyalkyl ether alcohols with high polarity can adsorb on the silica surface, thus weakenig the silica agglomerate and the filler–filler network. Silane coupling agents of this type include 3-mercaptopropyloxy-ethyoxyl-*bis*(tridecyl-pentaethoxy) siloxane (Si363) [21] and 3-mercaptopropyloxy-methyoxyl-*bis*(nonane-pentaethoxy) siloxane (Si747) [22]. Si363 and Si747, as water-soluble silane coupling agents, hydrolyze easily in water and modify silica directly in its solution state. The modified silica/rubber composite, with high scorch resistance, has higher processing safety as a result. Si747 has a significant cost advantage compared to the imported product Si363.

In this article, we explored the mechanism of interaction between Si747 and monodisperse or precipitated silica. Other researchers reported that the amount of silane coupling agent [12] and modification conditions [23,24] (temperature, pH) were important for the degree of silica surface modification. In particular, the hydrolysis and condensation reaction of the silane coupling agent is affected by the structure of the hydrolysis group, the reaction medium and the reaction conditions (temperature, pH, concentration, amount of water and catalyst) [25]. Pantoja [26] proved that pH has a great influence on the hydrolysis reaction of silane coupling agent γ-methacryloxypropyltrimethoxysilane (MPS) via infrared spectroscopy. Rostami [27] studied the effect of different pH on the surface chemistry of fumed silica modified by aminopropyltrimethoxysilane (APTMS). In this research study, we confirmed the optimum amount of Si747 and modified pH in terms of modification efficiency of AS and PS, respectively, using Fourier transform infrared (FT-IR) and thermal weight loss analysis (TGA), after which silica/ESBR master batches were prepared via the latex compounding technique. Finally, the properties of the silica/ESBR composites were investigated.

## 2. Materials and Methods

### 2.1. Materials

*l*-lysine (98%) was purchased from Aladdin Industrial Corporation (Shanghai, China); Ttraethoxysilane (TEOS, 98%) was produced by Sinopharm Chemical Reagent Co., Ltd. (Shanghai, China); Si747 was produced by Shanghai Cheeshine Chemicals Co., Ltd. (Shanghai, China); Commercial ESBR1502 latex with 23.32% mass fraction dry rubber content was produced by Sinopec Qilu Petrochemical Co., Ltd. (Zibo, China). Commercial PS (1165 MP, BET surface area 165 m^2^/g) was purchased from Solvay white carbon black of Qingdao Co., Ltd. (Qingdao, China). All of the rubber ingredients were industrial grade and used as received.

### 2.2. Preparation of Modified Silica

Specific amounts of AS (50 g) with desired sizes (28 nm, BET surface area 169 m^2^/g) were obtained via the method reported by Yokoi [28], using *l*-lysine as the catalyst. The synthetic silica suspension was concentrated to a volume of 150 mL using a rotary evaporator and reserved. As control, 50 g carefully weighed PS was added into the same volume (150 mL) of deionized water and stirred at 300 rpm for 30 min before use.

In our typical experiments, hydrochloric acid, saturated sodium bicarbonate solution and saturated sodium hydroxide were used to adjust the pH values to 3, 7, 9, and 12, respectively. Different masses of Si747 (mass ratio of Si747 to water = 11.11% and mass ratios of Si747 to silica = 8%, 10%, 12%, 15%, 20%, respectively) were added into deionized water for hydrolysis for 12 h at room temperature.

The pre-prepared AS suspension and PS slurry were then mixed with different amounts of Si747 hydrolysates. The volumes of all these mixed solutions were no more than 250 mL, and were stirred at 600 rpm and 80 °C for 6 h in an oil bath. For convenience of description, the modified silica solutions are denoted 8%-AS, 10%-AS, 12%-AS, 15%-AS, 20%-AS, 10%-PS, 12%-PS, 15%-PS, 20%-PS, 15%-AS@3, 15%-AS@7, 15%-AS@9, 15%-AS@12, 20%-PS@7, and 20%-PS@9.

Part of the pure silica and modified silica suspension were placed into a drying oven under 110 °C for 12 h. Silica powders were extracted in a Soxhlet extractor using ethanol for 24 h (110 °C, 30 min for each reflux) to remove the self-condensed Si747. Then, all silica powders after extraction were put into a vacuum drying oven at 65 °C for 24 h.

### 2.3. Preparation of Silica/ESBR Master Batches

Different types (monodisperse, precipitated, modification at different conditions) of silica solutions were agitated for 10 min using a mechanical stirrer and blended with the weighed ESBR latex. After stirring together for 30 min at 300 rpm with a mechanical stirrer, the master batches of silica/ESBR were co-flocculated using calcium ethylate (3 g calcium nitrate in 97 g ethyl alcohol). The flocculates were made to sheets manually and the above steps were repeated until the latex was completely demulsified. All master batch sheets were washed with water for several times. The residual solution was centrifuged and the solids and master batch sheets were then collected to dry at 55 °C in the oven until a constant weight was reached.

### 2.4. Preparation of Silica/ESBR Compounds and Vulcanizates

In order to obtain well-dispersed silica/ESBR compounds, two stages of mixing were carried out. First, silica/ESBR master batches were masticated for 3 min in a torque rheometer (RM-200C, Harbin Harper Electric Technology Co., Ltd., Harbin, China) for initial mixing, for which the initial temperature was set at 90 °C and the rotational speed was constant at 600 rpm. When the torque curve was stable, the compression lever was lifted. Following the formulation listed in Table 1, zinc oxide and stearic acid were added at the same time. After mixing for 8 min, the torque curve was stable and the temperature of the chamber reached 130 °C; the compounds were then taken out.

Next, *N*-*tert*-butylbenzothiazole-2-sulfonamide (NS), diphenyl guanidine (DPG) and sulfur (S) were successively added into the cooled compounds on a 6-inch two-roll mill (Dongguan Bolon Precision Testing Machines Co., Ltd., Dongguan, China), blending uniformly at room temperature. Condensate water was flowed into the roller constantly to maintain a temperature below 55 °C. The whole mixing process took 15 min for each sample and compound sheets with an approximate thickness of 2 mm were obtained.

The silica/ESBR compounds are denoted 8%-AS-R, 10%-AS-R, 12%-AS-R, 15%-AS-R 20%-AS-R, 10%-PS-R, 12%-PS-R, 15%-PS-R, 20%-PS-R, 15%-AS@3-R, 15%-AS@7-R, 15%-AS@9-R, 15%-AS@12-R, 20%-PS@7-R, and 20%-PS@9-R, based on the silica type and modification conditions.

The scorch time (t_s2_) and optimum cure time (t_90_) of the silica/ESBR compounds were measured using a rheometer vulcanization machine (MDR-2000, Alpha, Akron, OH, USA) at 160 °C after being stored at room temperature for 12 h. The oscillating frequency was 1.7 ± 0.1 Hz, with an amplitude of ±3°. The volume of each test specimen was 5 cm^3^. The cure rate index (CRI) was calculated using the following equation:

CRI (min^−1^) = 100/(t_90_ − t_s2_), (1)

The compounds were vulcanized at 160 °C and 10 MPa for (t_90_ + 2) min in a hydraulic press (HS100T-RTMO, Shenzhen Jiaxin Co., Ltd., Shenzhen, China).

### 2.5. Characterization

SEM photographs of the silica suspensions were taken on a Quanta FEG250 field emission scanning electron microscope (FEI Co., Ltd., Portland, OR, USA) to distinguish silica morphology between AS and PS before adding them to the rubber latex. Silica suspensions were dropped on silica wafers and sputter-coated with a thin layer of gold after drying at room temperature, to prevent electrical charging during examination. The measurement was performed at an accelerating voltage of 15 kV and operation distance was 10 mm.

The particle size of the silica suspensions was measured with a dynamic light scattering (DLS) instrument (Mastersizer 2000, Malvern Co., Ltd., Malvern city, UK), corresponding with the characterization by SEM. A trace of emulsifier (OP-10) was of great use to make silica particles discrete during testing.

The difference in reactive groups between pure and modified silica was identified on a Tensor 27 FTIR spectrometer (Bruker Co., Ltd., Ettlingen, Germany). Amounts of 10 mg of pure and modified silica powder were added into the mortar and ground with dried KBr powder; the mass ratio of silica to KBr was 0.0125. Infrared absorption tests were performed at the wavelength range of 400–4000 cm^−1^ and 32 scans were conducted.

Silica weight loss was measured on a TG209 thermo-gravimetric analyzer (NETZSCH Co., Ltd., Selb, Germany) under nitrogen atmosphere. The samples were heated from 30 to 850 °C at a heating rate of 10 °C/min.

The dynamic rheological properties of the silica/ESBR compounds were analyzed on a RPA2000 rubber process analyzer (Alpha Technologies Co., Ltd., Akron, OH, USA) at 60 °C. The strain sweep amplitude varied from 0.2 to 100% at the test frequency of 1 Hz. The curves of the storage modulus (G′)-strain (ε) were obtained.

The dynamic viscoelastic properties of the silica/ESBR vulcanizates were measured on a TQ800 (TA Co., Ltd., New Castle, DE, USA) in tension mode. The temperature was varied from −80 to 80 °C at a heating rate of 3 °C/min. The test frequency was 10 Hz and the strain amplitude was 0.2%. The loss angle tangent (tanδ) was measured as a function of temperature for samples under identical conditions. Long striped specimens were prepared from dumbbell-shaped specimens and both ends were cut off.

The physical–mechanical properties of the silica/ESBR vulcanizates, such as the tensile properties including the tensile strength, modulus at 300% elongation and elongation at break (ASTM D410), were determined using an Electrical Tensile Tester (AT-7000S, ZWICK Co., Ltd., Ulm-Einsingen, Germany) at a tensile rate of 500 mm/min. The specimens were prepared to a dumbbell shape that was punched out from a molded sheet. Five specimens were measured for each sample, and the average values were calculated and reported.

The silica dispersion in silica/ESBR vulcanizates was observed under a JSM-7500F scanning electron microscope (JEOL Co., Ltd., Shoshima City, Japan) with an accelerating voltage of 5 kV. The vulcanized rubber strips were frozen in liquid nitrogen and broken in their brittle state, then adhered to the conductive adhesive directly with the broken section exposed, and finally sprayed with gold in a vacuum environment.

## 3. Results

### 3.1. Characterization of Pure and Modified Silica

#### 3.1.1. SEM and DLS Results of Pure Silica

Based on our previous study [17], the morphology and size of the silica particles added to ESBR latex were measured using SEM and DLS, respectively. As shown in Figure S1, AS showed a morphology of spherical particles with an average size of 28 nm. In contrast, PS exhibited a broad distribution of particle sizes due to severe aggregation.

#### 3.1.2. Silane Coupling Agent Si747

**Figure 1:**
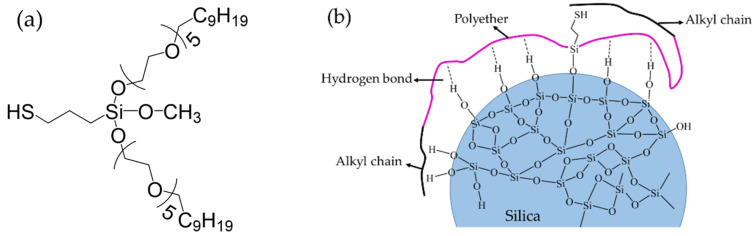
(**a**) Chemical structure of silane coupling agent Si747 and (**b**) schematic diagram of Si747 attached to the silica surface.

Figure 1a shows the chemical structure of the water-soluble silane coupling agent Si747. The Si atom is connected to a mercaptopropyl, of which the free mercapto group can be regarded as an active point to couple with the rubber chain in the vulcanization process. In addition, a methyoxyl group connected to the Si atom hydrolyzed to form Si–OH in contact with water, then dehydrated and condensed with the Si–OH on the surface of the silica, resulting in being chemically grafted onto silica surface. Subsequently, two long chains of polymeric substituents replace the volatile and easily hydrolysable methyoxyl group, which leads to the reduced emission of VOC. The polymeric substituents contain a polar polyether part and a hydrophobic alkyl part. The polyether part with its polar property ensures high silica affinity and fast adsorption and reaction on the silica surface, which compensate for the steric hindrance effect caused by the excessive volume of the substituent. Meanwhile, the alkyl part derived from olefin polymerization are at the end of the long polymeric substituents. On the one hand, the alkyl can shield the free mercapto group and delay the activating reaction of Si747, resulting in improved anti-scorch performance of silica/rubber compounds; on the other hand, the extra alkyls also shield the silanol group, leading to excellent hydrophobation of the silica. This weakens the interaction between the silica particles and improves dispersion. As depicted in Figure 1b, the long-chain nature of the polymeric substituents provides a special shielding effect.

**Figure 2:**
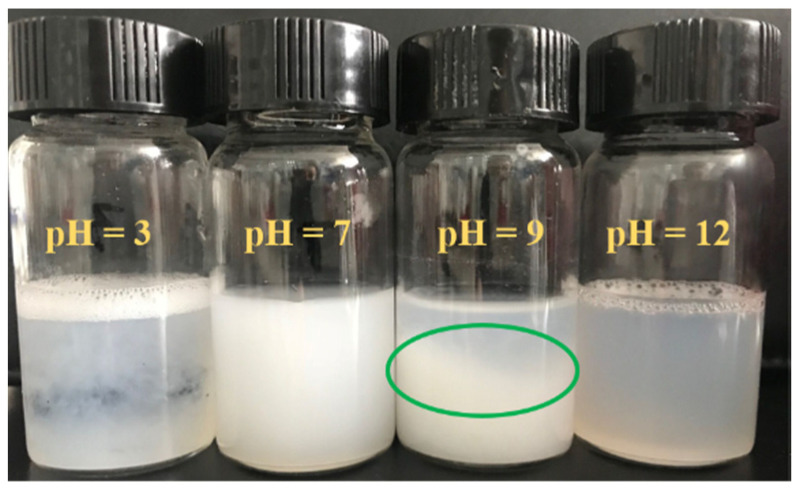
Pictures of Si747 hydrolyzed for 12 h at different pH and 30 °C.

Figure 2 shows the solution state of Si747 hydrolyzed for 12 h at different pH and a constant temperature of 30 °C. As we all know, there exists Si–OCH_3_ in the structure of Si747, which is easy to hydrolyze [29,30]. However, the condensation rate of the hydrolysates depends on the acid concentration, oxyhydrogen anion concentration, and the structure around the Si747 hydrolysis groups in the solution system [29]. Therefore, the pH value of the solution is the key parameter for controlling the relative rate and range of the competing process, which is the hydrolysis and condensation of the silane coupling agent [31]. As shown in Figure 2, at neutral pH (pH = 7), the whole hydrolysis solution showed a white turbid state, which was basically maintained as the initial state as Si747 was added into the solution, indicating that hydrolysis and condensation were weak. In a low pH (acid, pH = 3) solution, white floc presented, which was the self-condensate products formed by the hydrolysis and condensation of Si747. By comparison, in an alkaline environment, the whole solution system became transparent under strong alkaline (pH = 12) conditions; under weak alkaline conditions (pH = 9), the top layer of solution presented transparent and the bottom layer remained a white turbid substance, which was the unhydrolyzed Si747. All this indicates that Si747 could hydrolyze under alkaline conditions, and the rate of hydrolysis in a weak alkaline environment was slower than that in a strong alkaline condition [29]. All hydrolysates incurred no obvious condensation under alkaline conditions, and no self-condensate products formed, which may be related to the chemical environment of Si–OCH_3_ changed via the two long-chain polymeric substituents. Overall, Si747 had high hydrolysis and condensation rate in a strong acid system [31], while the lowest hydrolysis rate was obtained at neutral pH [29]. High hydrolysis and slow condensation rate were obtained under a strong alkaline condition [29], while moderate hydrolysis and condensation rate were obtained under a weak alkaline condition (pH = 9).

#### 3.1.3. FTIR Spectra of Pure and Modified Silica

**Figure 3:**
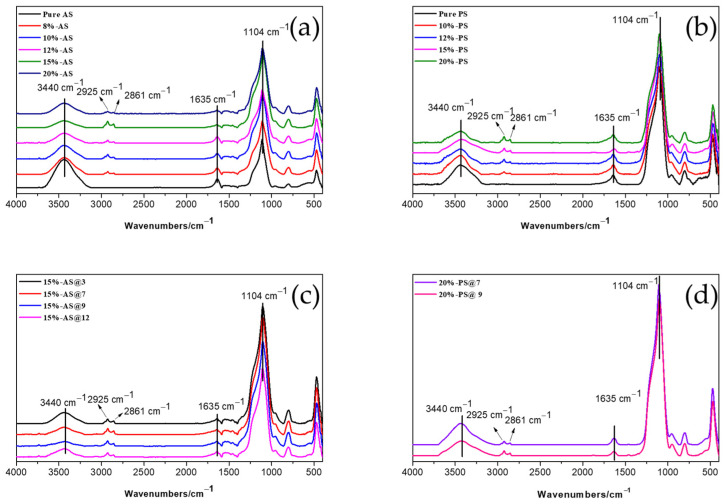
FTIR spectra of pure silica and silica modified with Si747: (**a**) AS; (**b**) PS; (**c**) AS modified at different pH; (**d**) PS modified at different pH.

The FTIR spectra of the two types of silica modified with different dosages of Si747 after rinsing are shown in Figure 3. It could be seen that the absorption peaks at 3440 and 1635 cm^−1^ corresponded to the stretching and deforming vibration modes, respectively, of the H–O–H bonds in the adsorbed water [32]; the absorbance ranging from 1000 to 1150 cm^−1^ was assigned to the Si–O–Si asymmetric stretching mode. Comparing the FTIR spectra of modified silica with pure silica, all modified silica had adsorption peaks at 2925 and 2861 cm^−1^ in the spectra curves, which were attributed to the vibrations of –CH_2_– and –CH_3_– bonds [24,33]. These –CH_2_– and –CH_3_– bonds that derive from Si747 were detected after rinsing, which proved that Si747 was successfully grafted onto the silica surface.

Figure 3c,d exhibit the FTIR spectra of the two types of silica modified with Si747 at different pH and constant 80 °C after rinsing.

It is well known that the relative intensity (RI) of the peak at 3440 cm^−1^ is determined by the number of –OH bonds [24,33]. Therefore, the higher the RI of the peak at 3440 cm^−1^, the greater the number of hydroxyl groups on the silica surface. Shown in Table 2 are the RI values, which were normalized to the calculated 3440 cm^−1^ peak in the FTIR spectra of the AS and PS before and after modification, respectively. It can be seen that the RI of the pure silica was higher than that of the silica modified with Si747. This is due to the chemical grafting and physical shielding of the silanol on the silica surface. For the modified AS, as the dosage of Si747 increased from 8% to 20%, the RI of the peak at 3440 cm^−1^ first decreased, then increased. The minimum value was reached when the amount of Si747 was 15%. As the amount of Si747 continued to increase to 20%, the RI value increased inversely. This is because during the hydrolysis process of Si747, self-condensation proceeded at the same time, and the degree of self-condensation increased as the dosage of Si747 increased. These self-condensates could sink into the aqueous system as precipitates, or be physically adsorbed on the silica surface and prevent the hydrolyzed Si747 contacting the silanol, resulting in the increased RI of the peak at 3440 cm+ after the adsorbed Si747 was rinsed off with toluene. For PS, the RI of the peak at 3440 cm^−1^ continued to decrease as the amount of Si747 increased. The minimum value was reached with 20% Si747. This may be because the surface activity of PS was higher than that of AS, and aggregation was more serious. A larger amount of Si747 was required.

Moreover, both the modified AS and PS presented the lowest RI of the peak at 3440 cm^−1^ at pH = 9, indicating that the silanol density on the surface was the lowest and the modification effect was optimal.

#### 3.1.4. TGA Analysis of Pure and Modified Silica

**Figure 4:**
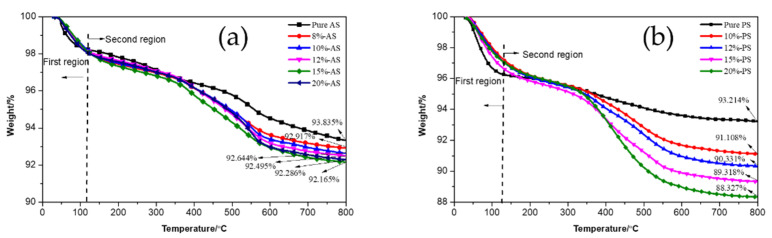

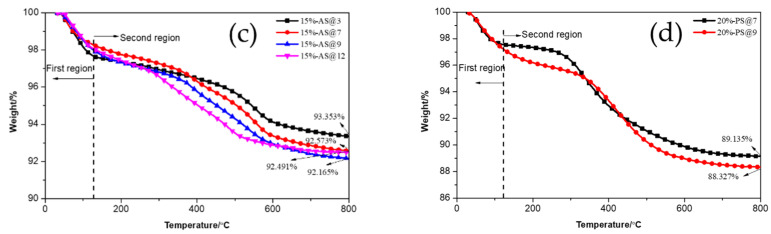
TGA curves of pure silica and silica modified with Si747: (**a**) AS; (**b**) PS; (**c**) AS modified at different pH; (**d**) PS modified at different pH.

Figure 4 shows the weight loss of pure and modified silica with different amounts of Si747 and at different pH after rinsing. It can be seen from the figures that over the process of temperature change, the weight loss curves of silica can be divided into two regions. In the first region, where the temperature was below 125 °C, the weight loss on the thermogravimetric curves was mainly due to the loss of adsorbed water on the silica surface. It should be noted that the weight loss in pure silica was higher than that in modified silica in this region, which indicates that the amounts of silanol and adsorbed water on the modified silica surfaces decreased. As the temperature increased in the second region, 125–800 °C, there was greater weight loss relative to the first region. The weight loss in pure silica was mainly the dehydroxylation of silica surface silanol, while that in the modified silica was not only the dehydroxylation of unreacted silanol but also the thermal decomposition of grafted Si747. For modified PS, the weight loss increased as the Si747 dosage increased, and the maximum value was reached with 20% Si747. For AS, weight loss was not very different with different Si747 amounts. However, as the amount of Si747 continued to increase, weight loss first increased, then decreased. Weight loss reached its maximum value with 15% Si747. These results were consistent with the RI pattern in Table 2. In conclusion, modified AS with 15% Si747 and PS with 20% Si747 exhibited the best effects. In addition, comparing the residual mass of the two types of modified silica with the optimum amount of Si747 at 800 °C indicated that PS had a higher degree of Si747 grafting.

For modified PS, with the increase in pH from 7 to 9, weight loss increased from 10.865% to 11.673%, indicating that the degree of Si747 grafting in modified PS was higher at pH = 9. For modified AS, weight loss was still not very different with different pH. However, it was still observed that as the pH continued to increase, the weight loss of silica first increased and then decreased. The maximum value was reached at pH = 9.

### 3.2. Characterization of ESBR/Silica Compounds

#### 3.2.1. Vulcanization Properties of ESBR/Silica Compounds

It is known that the vulcanization properties of ESBR/silica compounds determine their prospects for application.

Table 3 shows the vulcanization properties of ESBR compounds filled with different types of silica. The scorch and optimum curing time of ESBR/silica compounds are represented by t_s2_ and t_90_, respectively. The minimum torque M_L_ reflects the plasticity of compounds at a given temperature, and the maximum torque M_H_ reflects the modulus of the vulcanizates; M_H_–M_L_ indicates the maximum crosslink density of the composites. As the amount of Si747 increased, scorch resistance weakened, which was indicated by the shortened t_s2_; the shortened optimum curing time and increased curing rate indicated that the vulcanization process could be promoted by introducing Si747; the decreased M_L_ meant improved processability. Given the same amount of Si747, the scorch time of ESBR/MAS compounds was slightly shorter than that of ESBR/MPS compounds, which showed that the scorch resistance of the former was slightly weaker than that of the latter. Meanwhile, the curing rate of the former was slower than that of the latter according to the trend in the CRI values. Moreover, when comparing M_L_ values, the lower M_L_ values for the ESBR/MAS compounds indicated better processability. As the amount of Si747 increased, the crosslinking density of ESBR/MAS vulcanizates first increased, then decreased, reaching a maximum with 15% Si747, while, for ESBR/MPS vulcanizates, the crosslinking density continued to increase and reached a maximum with 20% Si747. It is worth noting that the crosslinking densities of the ESBR/MPS vulcanizates were greater than those of the ESBR/MAS vulcanizates for the same amount of Si747.

For ESBR/MAS compounds, as pH increased from acidic to alkaline, anti-scorch ability reduced as t_s2_ decreased. The optimum curing time t_90_ was shortened and the curing rate obviously increased. All these reached their maximum values at pH = 12. It is known that there is a large amount of hydroxyl groups on the silica surface, which causes the silica to be acidic and promotes the adsorption of alkaline accelerators (such as DPG) to delay vulcanization [1]. The AS was modified with Si747 at pH = 3 and blended with ESBR latex to co-flocculate. The compound system continued to be acidic and was not good for vulcanization. By contrast, as the pH increased, the basicity of the compound system gradually increased, which promoted the vulcanization process, including an increased curing rate and shortened scorch time. It should be noted that stronger alkalinity yielded inferior reinforcement of vulcanizates. Meanwhile, as pH increased from acidic to alkaline, the M_L_ values of the compounds and M_H_ and M_H_–M_L_ values of the vulcanizates all showed a trend of first decreasing, then increasing. This is because with the increase in pH, the degree of Si747 grafting on AS increased first, then decreased, reaching its maximum at pH = 9; as a result, as the degree of grafting increased, the silanol density on the silica surface decreased, the silica agglomerates weakened, the silica dispersion in the rubber matrix improved and the interaction between silica and rubber molecules was enhanced, which resulted in a decrease in the initial modulus M_L_ of the ESBR/silica compounds and an enhancement in processability. After vulcanization, all final moduli M_H_ and modulus differences M_H_–M_L_ in the vulcanizates increased. When pH continued to increase to 12, the grafting degree of Si747 on AS decreased, the particle agglomeration intensified, the M_L_ of compounds increased and plasticity was inferior as a result. Meanwhile, the modulus difference M_H_–M_L._ indicating the crosslinking density of vulcanizates, decreased.

For PS modified with Si747 at different pH values then filled with ESBR, the pattern of change in vulcanization characteristics was similar to AS.

#### 3.2.2. Dynamic Mechanical Properties of ESBR/Silica Compounds

**Figure 5:**
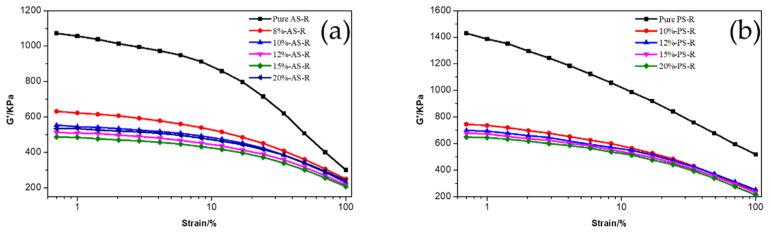

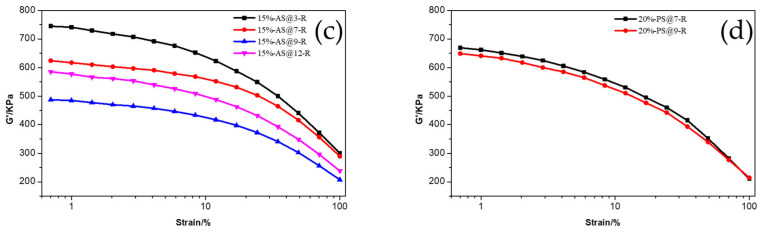
G′-Strain curves of ESBR compounds filled with different types of silica: (**a**) AS; (**b**) PS; (**c**) AS modified at different pH; (**d**) PS modified at different pH.

Figure 5 exhibits the G′-Strain curves of ESBR compounds filled with different types of silica modified with Si747. Tables S1–S3 show the shear storage modulus difference ΔG′ of ESBR compounds filled with AS and PS before and after modification, respectively.

In general, the phenomenon in which the shear storage modulus G′ of silica-filled rubber drops sharply with increasing strain is called the Payne effect. The difference in shear storage G′ between low strain and high strain can indicate the strength of the Payne effect, which reveals the dispersibility of the silica. The smaller the ΔG′, the weaker the Payne effect, and the better the dispersibility of the silica [34]. As shown in Figure 5, the shear storage modulus G′ and modulus difference ΔG′ of ESBR compounds filled with modified silica were lower than that of silica before modification and filling. The Payne effect of ESBR compounds filled with silica modified with Si747 was weakened and the dispersion of silica was improved. Meanwhile, the shear storage modulus difference ΔG′ of ESBR/AS compounds was always lower than that of ESBR/PS compounds in the corresponding state, which indicated that ESBR/AS compounds presented with weaker filler networks and Payne effects. The interactions between silica–silica particles were weak and the dispersion was good.

For ESBR/MAS compounds with AS modified with different amounts of Si747, as the amount of Si747 continued to increase, the degree of modification of monodisperse silica increased, while the initial modulus and ΔG′ of ESBR/MAS compounds gradually decreased, indicating that the Payne effect was weakened and the dispersion of silica was improved. When the amount of Si747 reached 15%, the modification degree of the silica surface was the highest and the initial modulus and ΔG′ of ESBR/MAS compounds were the lowest, indicating the weakest silica network and Payne effect along with the best relative filler dispersion.

For ESBR/MPS compounds, it is worth noting that as the amount of Si747 increased, the initial modulus and ΔG′ of ESBR/MPS compounds decreased continuously, indicating that the Payne effect and silica filler network were weakened and the silica dispersion was increasing. It reached optimal levels when the amount of Si747 was 20% by weight.

In summary, comparing AS with PS before and after modification when used to fill ESBR compounds, at constant amounts of Si747, ESBR/AS compounds had lower filler networks and Payne effects; therefore, AS dispersion was better.

In addition, as pH gradually increased, the initial modulus and ΔG′ of ESBR/MAS compounds with AS modified at different pH showed a trend of first decreasing, then increasing. The minimum values were reached at pH = 9.

It is worth noting that comparing ESBR/MAS compounds with AS modified with 15% Si747 at pH = 9 with PS modified with 20% Si747 at pH = 9, the initial modulus and ΔG′ of the former compounds were smaller than that of the latter.

### 3.3. Characterization of ESBR/Silica Vulcanizates

#### 3.3.1. Dynamic Viscoelastic Properties of ESBR/Silica Vulcanizates

**Figure 6:**
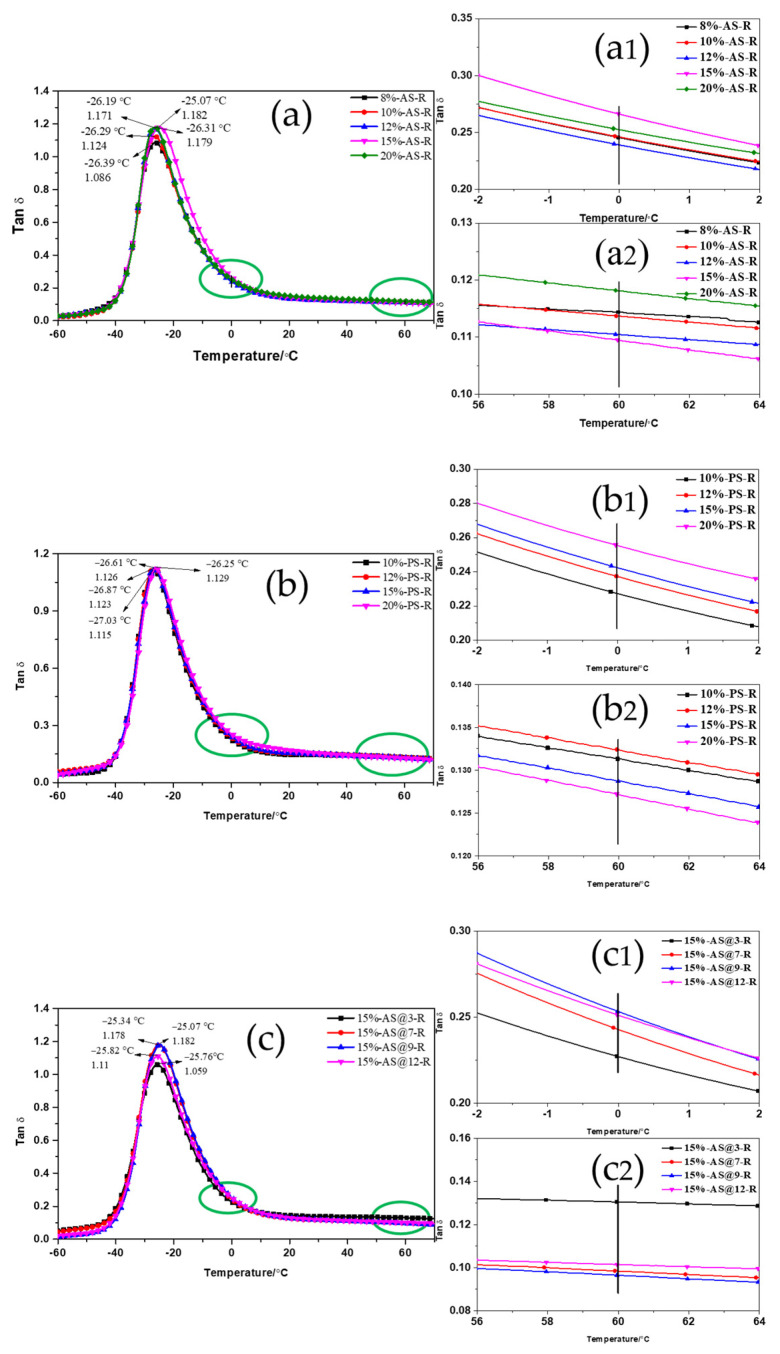

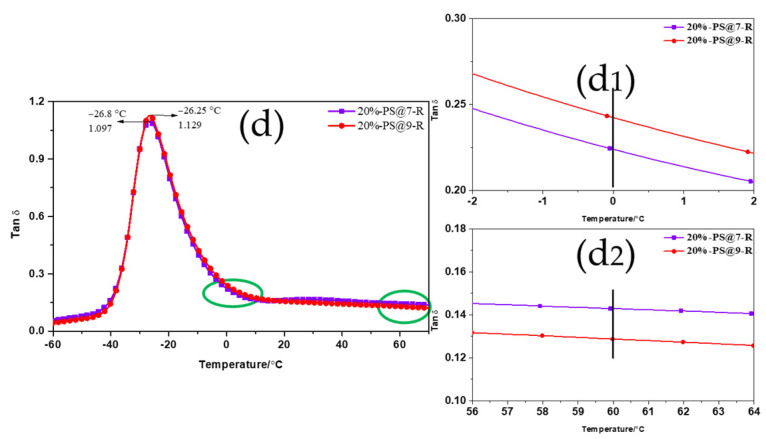
Temperature dependence of the loss factor of ESBR/MAS and ESBR/MPS vulcanizates: (**a**) AS; (**a1**) The tanδ at 0 °C; (**a2**) The tanδ at 60 °C; (**b**) PS; (**b1**) The tanδ at 0 °C; (**b2**) The tanδ at 60 °C; (**c**) AS modified at different pH; (**c1**) The tanδ at 0 °C; (**c2**) The tanδ at 60 °C; (**d**) PS modified at different pH; (**d1**) The tanδ at 0 °C; (**d2**) The tanδ at 60 °C.

The dynamic viscoelastic properties of ESBR/silica vulcanizates were tested through dynamic mechanical analysis. Figure 6 shows the temperature dependence of the loss factor of ESBR vulcanizates filled with AS and PS modified with different amounts of Si747. The loss factor (tanδ) first increased and then decreased with increasing temperature (T). The peak of tanδ-T curve represented the glass transition temperature (T_g_) for ESBR/silica vulcanizates. In Figure 6a, the ESBR/MAS vulcanizate with AS modified with 15% Si747 had the largest loss factor at T_g_; meanwhile, the T_g_ of this vulcanizate was the highest. Similarly, in Figure 6b, the ESBR/MPS vulcanizate with PS modified with 20% Si747 had the largest loss factor at T_g_ and the T_g_ was the highest. As indicated above, AS modified with 15% Si747 and PS modified with 20% Si747 displayed the best dispersion in the ESBR matrix.

The viscoelastic properties of tires, including wet skid resistance and rolling resistance, can be evaluated following the temperature dependence of the loss factor curve when ESBR/silica vulcanizate is applied to the tire tread. High-performance tires require a lower tanδ at 60 °C to reduce rolling resistance, and a higher tanδ at 0 °C to ensure high wet skid resistance. As shown in Figure 6, the ESBR/MAS vulcanizate with AS modified with 15% Si747 had the largest tanδ at 0 °C and the lowest tanδ at 60 °C, indicating that the dynamic viscoelastic properties of this vulcanizate were optimal. Meanwhile, the ESBR/MPS vulcanizate with PS modified with 20% Si747 had the best dynamic viscoelastic properties.

Observing the tanδ values in Tables S4 and S5, the wet skid resistance of ESBR/MAS vulcanizate with AS modified with 15% Si747 was enhanced by 4.27% compared with the ESBR/MPS vulcanizate with PS modified with 20% Si747; meanwhile, the rolling resistance was reduced by 13.92%.

As shown in Figure 6c,d, the vulcanizates exhibited the largest loss at the glass transition and the highest Tg when AS and PS were modified with Si747 at pH = 9, which indicated the silica with the best dispersion and strongest interaction with rubber. In addition, as shown in Table S6, ESBR vulcanizates filled with silica modified with Si747 at pH = 9 had the highest tanδ at 0 °C and lowest tanδ at 60 °C, which indicated that these vulcanizates had the optimal dynamic viscoelastic properties.

#### 3.3.2. Physical Mechanical Properties of ESBR/Silica Vulcanizates

As shown in Table 4, ESRB/MAS vulcanizate with AS modified with 15% Si747 exhibited the highest tensile strength, modulus at 300% elongation, reinforcing index and elongation at break. This performance in the mechanical properties of the vulcanizates was also due to the dispersion of silica, the interaction between silica and the rubber matrix, and the crosslinking density. Si747 could react with rubber through the mercaptopropyl group, thereby forming a “coupling bridge” between silica and rubber. The reinforcing efficiency of silica on rubber was enhanced via the increase of chemical interaction between silica and rubber [24]. Based on the data, AS modified with 15% Si747 had the maximum grafting degree (Figure 4); the modified AS-filled ESBR compounds had the best dispersion of silica and weakest Payne effect (Figure 5); the vulcanizate had the highest crosslinking density (Table 3). All of these contributed to reinforcing the rubber. Similarly, the ESBR/MPS compound with PS modified with 20% Si747 also presented the highest tensile strength, modulus at 300% elongation, reinforcing index and elongation at break.

It is noted that, according to Table 4 and Table 5, the ESBR/MAS vulcanizate exhibited lower hardness, modulus at 100% and 300% elongation, and reinforcing index compared to the ESBR/MPS vulcanizate at the same amount of Si747. All these may be related to the lower crosslinking density of the former compared to that of the latter, while the tensile strength and elongation at break of the former had obvious advantages over the latter. The tensile strength of ESBR/MAS vulcanizates with AS modified with 15% Si747 was enhanced by 14.8% compared to that of ESBR/MPS vulcanizates with PS modified with 20% Si747; meanwhile, elongation at break was enhanced by 62.4%, and hardness and modulus at 300% elongation were decreased by 13.1% and 53.2%, respectively.

The physical mechanical properties of the ESBR vulcanizates filled with two types of silica modified at different pH are shown in Table 6. For AS, with the increase in pH from acidic to alkaline, the tensile strength of the ESBR/silica vulcanizates obviously first increased, then decreased, reaching its maximum at pH = 9. Likewise, the modulus at 300% elongation, the reinforcing index and elongation at break of vulcanizates all presented the same tendency as the tensile strength as pH increased. These are mainly related to the chemical interaction between silica and rubber; that is, the crosslinking density of vulcanizates played an important role. At pH = 9, AS with the highest grafting degree of Si747, which can combine silica with rubber via chemical reaction, dispersed well in the rubber matrix and had the strongest interaction with the rubber molecule chain. In conclusion, the ESBR/MAS vulcanizates had excellent physical mechanical properties with the highest crosslinking density.

In contrast, the modulus at 100% elongation also reflected the ability of the vulcanizates to resist external force without deformation and exhibited a change tendency opposite from that of the modulus at 300% elongation. At this time, due to the low deformation rate of the vulcanizates, the filler network was the main contribution to the modulus. As modification efficiency increased, the silica dispersed better in the rubber, and the modulus at 100% elongation of vulcanizates decreased.

Compared to those at neutral pH, ESBR/MPS vulcanizates with PS modified at pH = 9 exhibited better physical mechanical properties, such as tensile strength, modulus at 300% elongation, reinforcing index and elongation at break.

#### 3.3.3. Micromorphology of ESBR/Silica Vulcanizates

**Figure 7:**
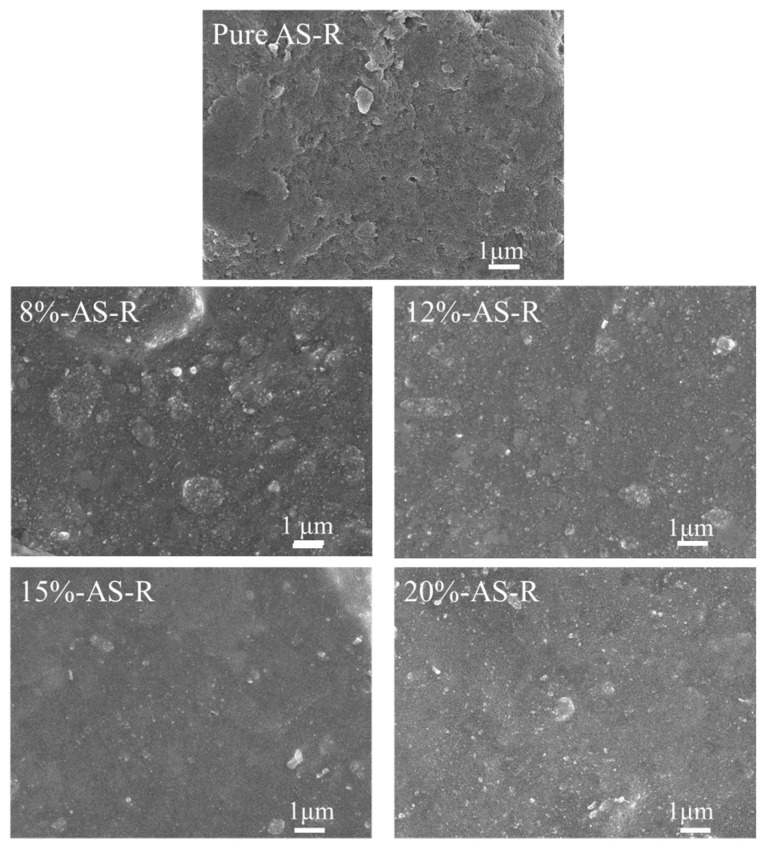
Fracture surface SEM micrographs of ESBR/AS vulcanizates before and after modification.

**Figure 8:**
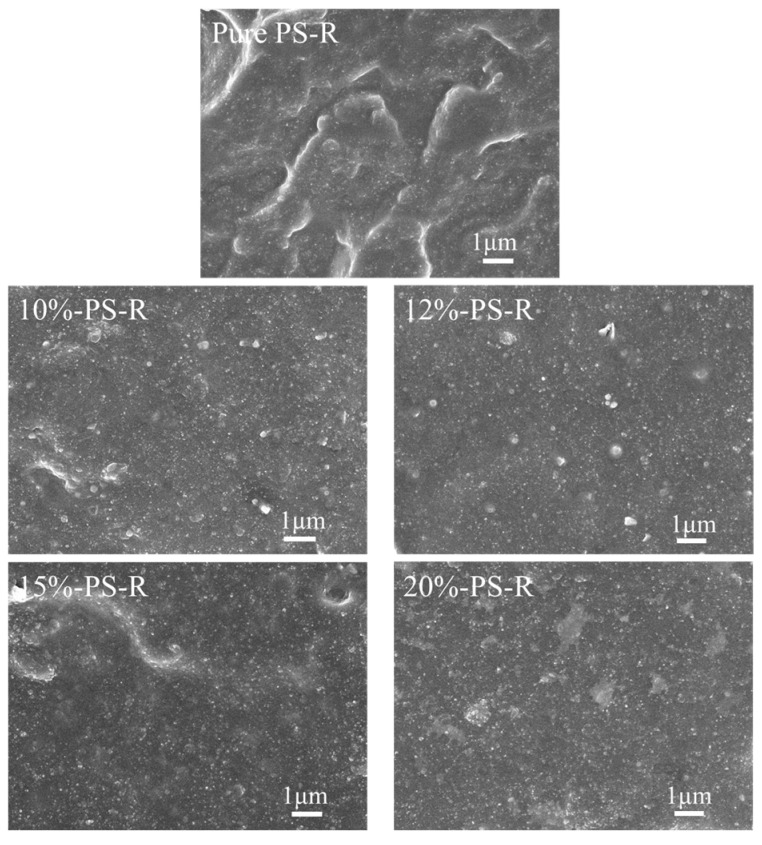
Fracture surface SEM micrographs of ESBR/PS vulcanizates before and after modification.

Figure 7 and Figure 8 show SEM images of the brittle fracture section of ESBR vulcanizates filled with different types of silica before and after modification. The bare white particles in the images are the silica under contrast via SEM. The strength of the interaction between silica and rubber can be determined by observing the amount of bare silica. In the process of brittle fracture, rubber molecules slipped off the surface of the silica and the fracture site should be a cross-linked network if the silica particles interacted strongly with the rubber molecules. If a rubber molecule separated from the silica surface, the silica would be exposed and distributed at the fracture section. As well, the more homogeneous the dispersion of silica, the better the comprehensive performance of the ESBR vulcanizate [23].

It is known that, as the grafting degree of Si747 increased, the dimension of silica aggregates decreased and the modified silica dispersed more uniformly in the rubber, enhancing the interaction between modified silica and rubber. As shown in Figure 7, as the amount of Si747 increased from 8% to 20%, the aggregation of silica in rubber matrix first weakened, then slightly strengthened. Meanwhile, the exposed amount of silica first decreased, then increased, and the minimum value was reached when 15% Si747 was used, where the silica interacted with the rubber molecules most strongly. Similarly, as shown in Figure 8, for the ESBR/PS vulcanizates, when 20% Si747 was used, the amount of exposed silica in the rubber matrix was minimal and silica was dispersed most uniformly.

**Figure 9:**
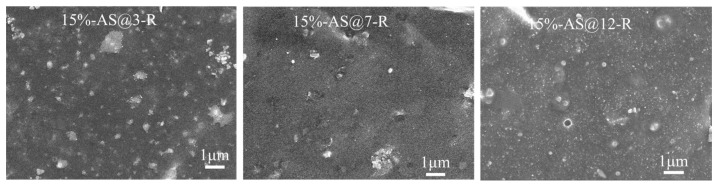
Fracture surface SEM micrographs of ESBR/MAS vulcanizates with AS modified at different pH.

Figure 9 and Figure S2 show SEM images of the brittle fracture sections of ESBR vulcanizates filled with AS and PS modified at different pH, respectively. In Figure 9, large AS aggregates can be observed clearly in the rubber matrix at pH = 3 and 7, implying inefficient modification and inferior dispersion of the silica. At a pH of 9 (shown in Figure 7 15%-AS-R), the vulcanizate had fewer silica aggregates and bare silica particles in comparison to the others. As the pH continuously increased above 9, more aggregates and bare particles appeared, indicating weaker interactions with rubber molecules.

For PS (Figure S2 and Figure 8 20%-PS-R), as the modification pH increased from 7 to 9, a small portion of rubber film existed at the fracture surface of the vulcanizate, implying that the interaction between the silica and the rubber matrix became stronger.

## 4. Conclusions

In this study, AS was successfully synthesized through the hydrolysis and condensation of tetraethoxysilane with L-lysine as the catalyst; the size and surface area of AS could be controlled similar to that of PS. Both AS and PS slurry could be modified using water-soluble 3-mercaptopropyloxy-methoxyl-bis(nonane-pentaethoxy) siloxane (Si747) directly and then blended with ESBR latex to prepare silica/ESBR master batches via the latex compounding technique. In this article, the effect of amount of Si747 and modification pH on silica and the comprehensive properties of the resulting silica/ESBR composites were systematically examined. The modification of silica was achieved by chemical condensation between hydrolyzed Si747 and silanol on the silica surface, along with physical absorption between a polar polyether in two long-chain polymeric substituents and silanol on the silica surface. FT-IR and TGA results for modified AS or PS powder showed that the optimum amounts of Si747 for AS and PS were 15% and 20%, respectively. Meanwhile, the optimum modification pH was 9. ESBR/MAS compounds presented weaker filler networks and stronger filler–rubber interactions than ESBR/MPS compounds with the silica modified under the optimum modification condition. Comparing the physical mechanical properties of the ESBR/MAS and ESBR/MPS vulcanizates, the tensile strength and elongation at break were enhanced by 14.8% and 62.4%, respectively, while the hardness and modulus at 300% elongation were decreased by 13.1% and 53.2%, respectively. Notably, for the dynamic properties, the ESBR/MAS vulcanizate exhibited a better combination of low rolling resistance with high wet skid resistance. Considering the performance of monodisperse silica filled ESBR composites, AS may be applied as an ideal substitution for PS in the green tire industry.

## Figures and Tables

**Table 1:** Formulation of Silica/ESBR Compounds.

Material	Content/phr ^1^
Dried ESBR ^2^	100
AS	50
PS	50
Si747	Variable ^3^
Zinc oxide ^4^	3
Stearic acid ^5^	1
*N*-*tert*-butylbenzothiazole-2-sulfonamide ^6^	1.5
Diphenyl guanidine ^7^	1.5
Sulfur ^8^	1.75

^1.^ Parts per hundred of rubber. ^2.^ ESBR latex was coagulated and dried, then weighed the mass. ^3.^ The amount of Si747 was calculated based on the mass of silica and the modification conditions could be variable. ^4.^ Zinc oxide was used to activate the whole vulcanization system and improve the crosslinking density and aging resistance of vulcanized rubber. ^5.^ Stearic acid can be used as plasticizer and is conducive to the full dispersion of silica, while reacting with zinc oxide to promote vulcanization. ^6.^ *N*-*tert*-butylbenzothiazole-2-sulfonamide was used as the after-effect vulcanization accelerator. ^7.^ Diphenyl guanidine was used as the medium speed vulcanization accelerator. ^8.^ Sulfur was used as the cross-linking agent.

**Table 2:** Relative intensities of pure silica and modified silica at the 3440 cm^−1^ peak.

**Sample**	**Pure AS**	**8%-AS**	**10%-AS**	**12%-AS**	**15%-AS**	**20%-AS**
RI ^1^/cm^−1^	0.242	0.1432	0.118	0.0844	0.0596	0.0896
**Sample**	**Pure PS**	**-**	**10%-PS**	**12%-PS**	**15%-PS**	**20%-PS**
RI/cm^−1^	0.0684	-	0.0653	0.0500	0.0459	0.0404
**Sample**	**15%-AS@3**	**15%-AS@7**	**15%-AS@9**	**15%-AS@12**	**20%-PS@7**	**20%-PS@9**
RI/cm^−1^	0.05961	0.03934	0.0243	0.04442	0.40098	0.27502

^1^ Relative intensity.

**Table 3:** Vulcanization characteristics of ESBR compounds filled with different types of silica.

Sample	Compound Types	t_s2_/min	t_90_/min	CRI/min^−1^	M_L_/dN·m	M_H_/dN·m	M_H_–M_L_/dN·m
Pure ESBR	-	1.04	4.66	27.62	0.29	4.13	3.84
ESBR/AS compounds	Pure AS-R	5.57	10.94	18.62	2.96	18.13	15.17
	8%-AS-R	1.77	6.43	21.46	1.85	13.81	11.96
	10%-AS-R	1.49	5.29	26.32	1.75	13.78	12.03
	12%-AS-R	1.43	4.01	38.76	1.72	13.94	12.22
	15%-AS-R	1.14	3.43	43.67	1.70	14.37	12.67
	20%-AS-R	0.91	3.09	45.87	1.51	13.28	11.77
	15%-AS@3-R	2.48	6.30	26.18	2.40	10.63	8.23
	15%-AS@7-R	1.31	3.98	37.45	2.18	11.21	9.03
	15%-AS@9-R	1.14	3.43	43.67	1.70	14.37	12.67
	15%-AS@12-R	1.00	2.85	54.05	2.31	13.48	11.17
ESBR/PS compounds	Pure PS-R	2.09	7.02	20.28	2.83	17.18	14.35
	10%-PS-R	1.59	3.52	51.81	2.18	14.27	12.09
	12%-PS-R	1.45	3.33	53.19	2.04	14.43	12.39
	15%-PS-R	1.19	2.69	66.67	1.94	14.54	12.7
	20%-PS-R	0.93	2.39	68.49	1.68	14.66	12.98
	20%-PS@7-R	1.51	3.72	45.25	1.89	13.02	11.13
	20%-PS@9-R	0.93	2.39	68.49	1.68	14.66	12.98

**Table 4:** Physical mechanical properties of ESBR/AS vulcanizates before and after modification.

Sample	Pure ESBR	Pure AS-R	8%-AS-R	10%-AS-R	12%-AS-R	15%-AS-R	20%-AS-R
Shore A/°	41	63	56	55	54	53	54
Tensile strength/MPa	1.90 ± 0.05	4.46 ± 0.06	10.88 ± 0.12	11.67 ± 0.15	12.42 ± 0.22	15.70 ± 0.52	13.33 ± 0.41
Modulus at 100% elongation/MPa	0.63 ± 0.02	1.41 ± 0.04	1.16 ± 0.03	1.11 ± 0.03	0.99 ± 0.02	1.09 ± 0.03	1.10 ± 0.03
Modulus at 300% elongation/MPa	1.13 ± 0.02	2.22 ± 0.05	2.63 ± 0.06	2.78 ± 0.06	2.82 ± 0.07	3.75 ± 0.08	3.64 ± 0.07
Reinforcing index	1.79	1.57	2.27	2.50	2.91	3.44	3.31
Elongation at break/%	520 ± 13	476 ± 12	583 ± 15	672 ± 22	687 ± 23	716 ± 28	664 ± 21

**Table 5:** Physical mechanical properties of ESBR/PS vulcanizates before and after modification.

Sample	Pure ESBR	Pure PS-R	10%-PS-R	12%-PS-R	15%-PS-R	20%-PS-R
Shore A/°	41	68	61	59	60	61
Tensile strength/MPa	1.90 ± 0.05	8.57 ± 0.08	10.69 ± 0.10	11.45 ± 0.15	11.95 ± 0.15	13.68 ± 0.20
Modulus at 100% elongation/MPa	0.63 ± 0.02	3.49 ± 0.06	1.55 ± 0.03	1.39 ± 0.04	1.41 ± 0.04	1.43 ± 0.03
Modulus at 300% elongation/MPa	1.13 ± 0.02	-	6.23 ± 0.08	7.02 ± 0.08	7.55 ± 0.08	8.20 ± 0.09
Reinforcing index	1.79	-	4.02	5.05	5.35	5.73
Elongation at break/%	520 ± 13	271 ± 8	374 ± 9	380 ± 9	438 ± 10	441 ± 10

**Table 6:** Physical mechanical properties of ESBR vulcanizates filled with AS and PS modified with Si747 at different pH.

Sample	15%-AS@3-R	15%-AS@7-R	15%-AS@9-R	15%-AS@12-R	20%-PS@7-R	20%-PS@9-R
Shore A/°	60	54	53	57	62	61
Tensile strength/MPa	11.47 ± 0.12	13.06 ± 0.22	15.70 ± 0.52	13.90 ± 0.25	12.76 ± 0.20	13.68 ± 0.20
Modulus at 100% elongation/MPa	1.46 ± 0.03	1.21 ± 0.03	1.09 ± 0.03	1.10 ± 0.03	1.54 ± 0.04	1.43 ± 0.03
Modulus at 300% elongation/MPa	3.30 ± 0.06	3.60 ± 0.05	3.75 ± 0.08	3.15 ± 0.06	7.02 ± 0.08	8.20 ± 0.09
Reinforcing index	2.26	2.98	3.44	2.92	4.56	5.73
Elongation at break/%	496 ± 15	630 ± 25	716 ± 28	608 ± 23	434 ± 12	441 ± 10

## Data Availability

Not applicable.

## Supplementary Materials

The following supporting information can be downloaded at: www.mdpi.com/article/10.3390/polym15040981/s1. Figure S1. SEM images and corresponding DLS results of silica particles: (a) AS; (b) corresponding DLS results of AS with single peak and average size of 28 nm; (c) PS; and (d) corresponding DLS results of PS with multiple peaks and average size of 370 nm. Figure S2. Fracture surface SEM micrographs of ESBR/PS vulcanizates with PS modified at different pH. Table S1. Shear storage modulus difference ΔG′ of ESBR compounds filled with monodisperse silica before and after modification. Table S2. Shear storage modulus difference ΔG′ of ESBR compounds filled with precipitated silica before and after modification. Table S3. Shear storage modulus difference ΔG′ of ESBR compounds filled with silica modified at different pH. Table S4. Tanδ at 0 °C and 60 °C of ESBR vulcanizates filled with monodisperse silica modified with different dosages of Si747. Table S5. Tanδ at 0 °C and 60 °C of ESBR vulcanizates filled with precipitated silica modified with different dosages of Si747. Table S6. Shear storage modulus difference ΔG′ of ESBR compounds filled with silica modified at different pH.
