# Supplementary material for: ESBR Nanocomposites Filled with Monodisperse Silica Modified with Si747: The Effects of Amount and pH on Performance
Source: Polymers (Basel). 2023 Feb 16;15(4):981. doi: 10.3390/polym15040981 (PMC9966922; doi:10.3390/polym15040981)
Supplement: Supplementary file 1 [file polymers-15-00981-s001.zip › polymers-2151993-supplementary.pdf]

# ESBR Nanocomposites Filled with Monodisperse Silica Modified with Si747: The Effects of Amount and pH on Performance

**Lijian Xia [1,2], Anmin Tao [1], Jinyun Cui [1], Abin Sun [1], Ze Kan [1,*] and Shaofeng Liu [1]**

[1] Key Laboratory of Biobased Polymer Materials, Shandong Provincial Education Department, School of Polymer Science and Engineering, Qingdao University of Science and Technology, Qingdao 266042, China
[2] State Key Laboratory of Marine Coatings, Marine Chemical Research Institute Co., Ltd., Qingdao 266072, China
* Correspondence: zkan@qust.edu.cn

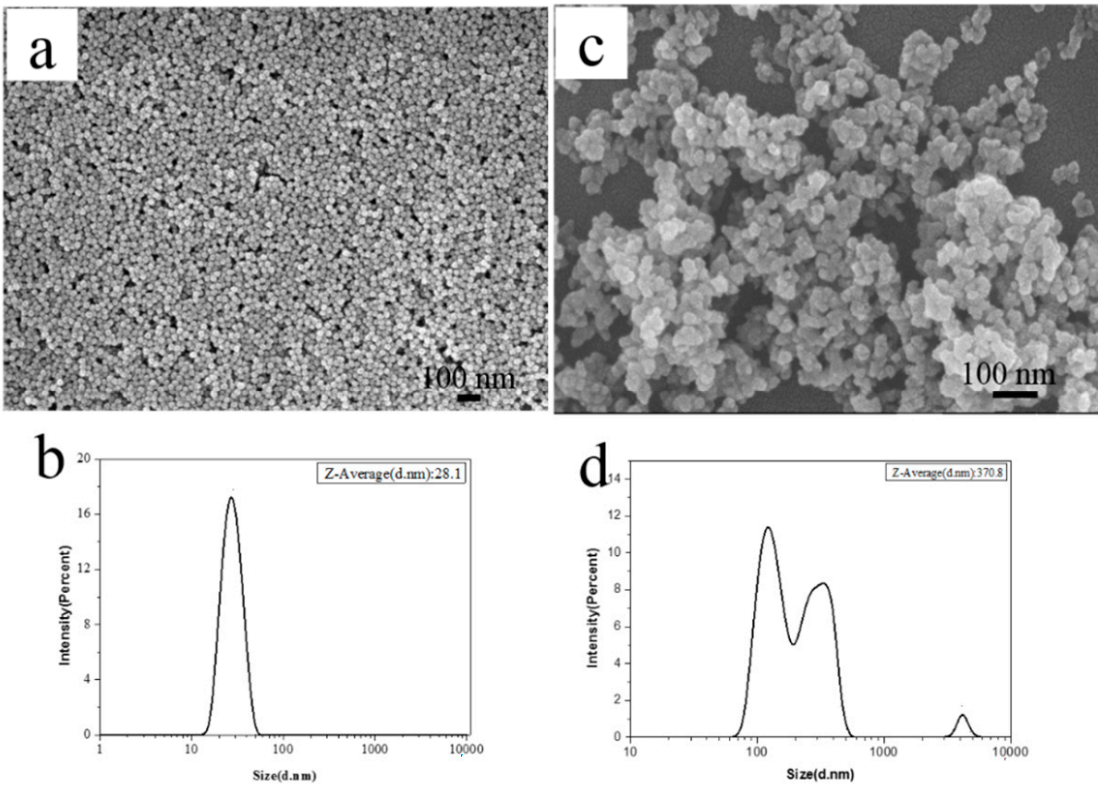


**Figure S1.** SEM images and corresponding DLS results of silica particles; (a) AS; (b) the corresponding DLS results of AS with single peak and average size of 28 nm; (c) PS; and (d) the corresponding DLS results of PS with multiple peaks and average size of 370 nm.

**Table S1.** The shear storage modulus difference Δ$G'$ of ESBR compounds filled with monodisperse silica before and after modification

| Sample | Pure AS-R | 8%-AS-R | 10%-AS-R | 12%-AS-R | 15%-AS-R | 20%-AS-R |
|---|---|---|---|---|---|---|
| Δ$G'$/KPa | 770.56 | 381.17 | 322.9 | 295.38 | 279.43 | 294.27 |

**Table S2.** The shear storage modulus difference Δ$G'$ of ESBR compounds filled with precipitated silica before and after modification

| Sample | Pure PS-R | 10%-PS-R | 12%-PS-R | 15%-PS-R | 20%-PS-R |
|---|---|---|---|---|---|

| | | | | | |
|---|---|---|---|---|---|
| Δ$G'$/KPa | 911.29 | 508.84 | 446.46 | 445.61 | 435.28 |

Table S3. The shear storage modulus difference Δ$G'$ of ESBR compounds filled with silica modified at different pH

| Sample | 15%-AS @3-R | 15%-AS @7-R | 15%-AS @9-R | 15%-AS @12-R | 20%-PS @7-R | 20%-PS @9-R |
|---|---|---|---|---|---|---|
| Δ$G'$/KPa | 445.1 | 334.81 | 279.43 | 347.49 | 458.95 | 435.28 |

**Table S4.** Tanδ at 0 °C and 60 °C of ESBR vulcanizates filled with monodisperse silica modified by different dosage of Si747

| Sample | 8%-AS-R | 10%-AS-R | 12%-AS-R | 15%-AS-R | 20%-AS-R |
|---|---|---|---|---|---|
| Tanδ@0 °C | 0.2455 | 0.2460 | 0.2391 | 0.2662 | 0.2524 |
| Tanδ@60 °C | 0.1144 | 0.1137 | 0.1105 | 0.1095 | 0.1181 |

**Table S5.** Tanδ at 0 °C and 60 °C of ESBR vulcanizates filled with precipitated silica modified by different dosage of Si747

| Sample | 10%-PS-R | 12%-PS-R | 15%-PS-R | 20%-PS-R |
|---|---|---|---|---|
| Tanδ@0 °C | 0.2272 | 0.2370 | 0.2424 | 0.2553 |
| Tanδ@60 °C | 0.1313 | 0.1323 | 0.1287 | 0.1272 |

**Table S6.** The shear storage modulus difference Δ$G'$ of ESBR compounds filled with silica modified at different pH

| Sample | 15%-AS @3-R | 15%-AS @7-R | 15%-AS @9-R | 15%-AS @12-R | 20%-PS @7-R | 20%-PS @9-R |
|---|---|---|---|---|---|---|
| Tanδ@0 °C | 0.2270 | 0.2426 | 0.2533 | 0.2510 | 0.2238 | 0.2423 |
| Tanδ@60 °C | 0.1405 | 0.1214 | 0.1095 | 0.1112 | 0.1429 | 0.1287 |

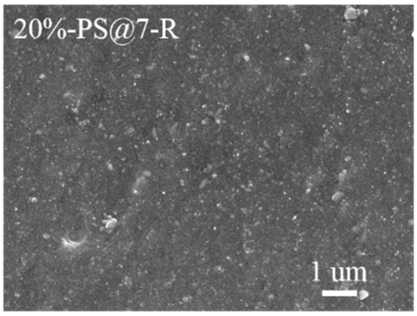


**Figure S2.** Fracture surface SEM micrographs of ESBR/PS vulcanizates with PS modified at different pH
